# Steady-state pharmacokinetics of lamivudine in end-stage kidney failure persons with detectable and undetectable HIV-1 RNA in peritoneal dialysis effluent

**DOI:** 10.1186/s40001-024-01972-8

**Published:** 2024-07-18

**Authors:** Teboho Mooko, Feziwe Busiswa Bisiwe, Enkosi Mondleki, Molefi Daniel Morobadi, Perpetual Chikobvu, Martin Munene Nyaga, Asis Bala, Dominique Goedhals, Thabiso Rafaki Petrus Mofokeng, Gabre Kemp, Kwazi Celani Zwakele Ndlovu

**Affiliations:** 1 Department of Internal Medicine (G73), University of the Free State, PO Box 339, Bloemfontein, 9300 South Africa; 2 Next Generation Sequencing Unit and Division of Virology, University of the Free State, Bloemfontein, South Africa; 3 Division of Nephrology, University of the Free State, Bloemfontein, South Africa; 4 Department of Pharmacology, University of the Free State, Bloemfontein, South Africa; 5 Division of Virology, University of the Free State, Bloemfontein, South Africa; 6 Ampath Laboratories, Pretoria, South Africa; 7 Department of Health of the Free State, Bloemfontein, South Africa; 8 Department of Community Health, University of the Free State, Bloemfontein, South Africa; 9 Pharmacology and Drug Discovery Research Laboratory, Division of Life Sciences, Institute of Advanced Study in Science and Technology (IASST), Vigyan Path, Guwahati, Assam 781035 India; 10 PathCare Vermaak, Pretoria, South Africa; 11 Department of Microbiology and Biochemistry, University of the Free State, Bloemfontein, South Africa; 12 Kidney and Hypertension Research Unit and Division of Nephrology and Hypertension, University of Cape Town, Cape Town, South Africa

**Keywords:** Lamivudine, Pharmacokinetics, HIV shedding, Kidney failure, Peritoneal dialysis, Peritoneum

## Abstract

**Background:**

Renally adjusted lamivudine dosages are effective. However, some of the kidney failure patients managed with lamivudine-containing regimens are failing to suppress HIV in peritoneal dialysis (CAPD) effluent. The steady-state lamivudine pharmacokinetics among these patients was evaluated.

**Methods:**

This overnight open-label pharmacokinetic study enrolled participants living with HIV and managed with CAPD. Lamivudine levels in blood serum and CAPD effluent samples were quantified using liquid chromatography coupled with a mass spectrometer. Pharmacokinetic measures were obtained through non-compartmental analysis.

**Results:**

Twenty-eight participants were recruited with a median antiretroviral (ARV) drug duration of 8 (IQR,4.5–10.5) years and a CAPD duration of 13.3 (IQR,3.3–31.9) months. 14.3% (4/28) had detectable unsuppressed HIV-1 viral load in CAPD effluents. The majority (78,6%,22/28) of participants received a 50 mg dose, while 10.7% (3/28), and another 10.7% (3/28) received 75 mg and 300 mg dosages, respectively. Among those treated with 75 and 300 mg, 66.7% (2/3) and 33.3% (1/3) had detectable HIV-VL in CAPD, respectively. The peritoneal membrane characteristics and CAPD system strengths were variable across the entire study population. Lamivudine exposure was increased in blood serum (50 mg-AUC_0-24 h_, 651.3 ng/mL; 75 mg-AUC_0-24 h_, 677.84 ng/mL; 300 mg-AUC_0-24 h_, 3135.89 ng/mL) compared to CAPD effluents (50 mg-AUC_0-24 h_, 384.91 ng/mL; 75 mg-AUC_0-24 h_, 383.24 ng/mL; 300 mg-AUC_0-24 h_, 2001.60 ng/mL) among the entire study population. The C_max_ (50 mg, 41.5 ng/mL; 75 mg, 53.2 ng/mL; 300 mg, 199.1 ng/mL) and C_min_ (50 mg, 17.8 ng/mL; 75 mg, 16.4 ng/mL; 300 mg, 76.4 ng/mL) measured in serum were within the therapeutic levels.

**Conclusions:**

Steady-state lamivudine pharmacokinetic measures were variable among the entire study population. However, the total lamivudine exposure was within the therapeutic levels.

## Key Learning Points

- **What was known:** steady-state lamivudine pharmacokinetics in persons living with HIV (PLHIV) and managed with peritoneal dialysis (PD).
- **This study adds**: HIV-1 shedding into CAPD effluents is not necessarily contributed by reduced lamivudine dosages pharmacokinetics.
- **Potential impact:** Renally adjusted lamivudine dosages are effective even in patients shedding HIV-1 into CAPD effluents.

## Introduction

Lamivudine (3TC) is a nuclease reverse transcriptase inhibitor (NRTI) agent used in combination with other antiretroviral drugs [1]. It is recommended by the World Health Organisation as the first-and second-line antiretroviral therapy (ART) regimen in the management of HIV and Hepatitis B infection [2]. Lamivudine should always be used with other antiretroviral drugs to achieve more effective viral suppression and to prevent the development of resistance [1]. Because lamivudine is excreted in urine, dose adjustment is required in patients with renal impairment [3].

The altered lamivudine dosages have shown to be effective even in people having kidney failure (KF) and managed with continuous ambulatory peritoneal dialysis (CAPD) [4, 5]. In comparison to subjects with normal renal function, those with impaired renal function had higher peak concentrations in the serum (C_max_), longer terminal elimination half-lives (T_1/2_), and areas under the serum concentration–time curves (AUCs) [6, 7]; based on the results from these studies, lamivudine dose adjustment is required in KF patients. Dose reduction for renal insufficiency can be accomplished by altering either dose size or dosing interval [8]. The current local HIV treatment guidelines in persons managed with CAPD recommend daily dosing of an oral tablet or solution at 50 mg or 75 mg depending on the creatinine clearance, glomerular filtration rate, and tolerance [4].

After oral administration lamivudine has a rapid dissolution rate and a bioavailability of approximately 82% in adults; it takes roughly 0.5 to 1.5 h to reach maximum serum concentrations (C_max_) in the non-KF population [1]. However, in the KF population, the time of maximum concentration (T_max_) is delayed in patients receiving drugs such as phosphate binders that interact with its active metabolite, 5′-triphosphate, and may lower the bioavailability [9]. Approximately 70% of an oral dose of lamivudine is excreted unchanged by the kidneys in patients with normal renal function [1] and 16% is removed by CAPD [6] because of its low molecular weight and decreased serum protein binding capacity; furthermore, CAPD dwell duration, peritoneal membrane type, blood supply to the peritoneal cavity as well as the strength of dialysate solution may be possible inter-patient pharmacokinetic variability factors likely to contribute to variable lamivudine clinical effects and outcomes [9].

Lamivudine dosages of 10 mg/mL solution and 150 mg tablet in the KF population managed with dialysis were shown to have similar AUC_0-24_, C_max_, and longer T_1/2_ compared to populations with normal kidney function [5, 6]. However, shedding of HIV-1 into CAPD effluents has been suggested in patients who are on steady-state lamivudine concentration [10–13]. Thus, the aim of this study was to evaluate the steady-state lamivudine pharmacokinetics in KF cohorts of participants managed with CAPD and in relation to HIV-1 shedding in CAPD effluents.

## Materials and methods

### Ethics statement

The study protocol was approved by the University of the Free State-Health Sciences Research Ethics Committee (UFS-HSREC) (UFS-HSD2020/0318/2710 & UFS-HSD2021/0267/2505 & UFS-HSD2021/0267/2505-0006), and permission for the study was granted by the Free State Department of Health. All participants provided written informed consent before enrolment, and study procedures were done in accordance with the Helsinki Declaration of Clinical Research on Human subjects [14].

### Study population

This is a sub-study of the main prospective cross-sectional study [12]. It recruited people living with HIV and kidney failure and managed with an ART-lamivudine-containing regimen and CAPD at Universitas Academic Hospital. The eligibility criteria included participants who were 18 years and older at the time informed consent was signed and living with HIV on ART for more than 3 months. Participants were excluded if they had active peritonitis at the time of enrolment, had an ART treatment duration of less than 3 months, showed any significant hematologic, hepatic, or pancreatic dysfunction, any documented ART non-adherence and history of substance abuse.

### Study design

This was an open-label non-randomised pharmacokinetics study performed at one of the clinical research organisations (FARMOVs) at the University of the Free State main campus. Participants were invited for a three-day admission after their routine clinic visit day. On the 1st day of admission participants’ ART and CAPD prescriptions were assessed and recorded by the research nurse. Vital signs and anthropometric measurements were recorded, and laboratory data were derived from the electronic database of Universitas Academic Hospital. Y-sets, twin-bag systems, and conventional peritoneal dialysis (PD) solutions (Adcock Ingram and Fresenius, South Africa) were used in all CAPD patients. The ART dosages were according to the patient's local clinic prescription.

On the 2nd day of admission, a pre-dose sample of blood and CAPD effluent were taken 30 min before the morning or night exchange; CAPD exchange was completed to ensure the participant does not ‘dry-up’ and a regular ART prescription comprising of lamivudine dosage either one of 50, 75 or 300 mg was taken at 07:00 or 19:00 depending on the previous time of the participants’ daily dosage intake. On the 3rd day of admission, the ‘24-h’ samples were collected from each participant. During the entire admission period, participants were fed on a standardised’ renal-friendly’ diet.

6 mL serum samples were obtained at pre-dose, 0.5 h, and post-dose 1, 2, 4, 6, 8, 12, 16, and 24 h using a red-cap serum separation vacutainer (Lasec, South Africa). During this period, participants continued their usual CAPD schedule, four times post-dose as per the participants’ clinic CAPD prescription. Before each exchange, a CAPD effluent sample was collected using a sterile 10 mL syringe, directly from a functioning Tenckhoff catheter into a sterile specimen bottle. The first 10 mL of CAPD effluent was aspirated and discarded. All samples were transported on ice to the research laboratory, where whole blood was centrifuged at 2500 rpm for 20 min, and serum was separated in hooded benchtops. Both separated serum and aliquoted CAPD effluent samples were freeze-stored in 2 mL Eppendorf tubes at -70 °C throughout the study period.

### Quantification of lamivudine levels by LC–MS/MS

The lamivudine concentrations were measured using a validated LC–MS/MS method. Lamivudine and internal standard (abacavir-d4) in both serum and CAPD effluents were extracted, desalted, and concentrated onto methanol-conditioned C_18_ 1 mL solid phase extraction cartridges (Oasis Prime HLB 1 cc/30 mg, Waters) at a flow rate of 1 mL/min. The eluents were vacuum dried, reconstituted in a 500 µL H_2_O with 0.1% formic acid and separated on a C_18_ (50 mm × 2.0 mm, Aqua, Phenomenex) column and detected by positive-ion MRM (multiple reaction monitoring) using two transitions per analyte.

The nominal concentrations ranged from 0.005 to 1.25 ppm for the lamivudine calibration curve using 500 µL sample volume. The intraassay precision and accuracy of the method (coefficient of variation) were 9.1% and 10.6%, respectively, while at the lowest limit of quantification, it was 15.5% and 17.2%, respectively.

### Safety

The participants’ vital signs were checked during admission and monitored by research nurses during the course of this study; thereafter, by a nephrologist.

### Steady-state lamivudine pharmacokinetic analysis

The non-compartmental pharmacokinetic analysis was performed to estimate the standard pharmacokinetic parameters for lamivudine. The mean area under the time-versus-concentration curve from 0 till 24-h period (AUC_0-24 h_), half-life (T_1/2_), elimination constant rate (k_e_), as well as AUC from 0 till infinity (AUC_0-∞_) were extrapolated from the concentration–time curve using Stata version 15 (StataCorp LP, College Station, TX, USA). The AUC_0-∞_ was estimated using three different models, estimated with a linear fit, estimated with an exponential fit, and estimated with a linear fit of the natural log concentrations. Furthermore, the maximum concentration (C_max_) in serum and CAPD samples and the time of maximum concentration (T_max_) as well as trough levels before the next dosage (C_min_) were estimated from the concentration–time curve.

### Statistical analysis

Continuous variables were summarized as medians and interquartile ranges (IQRs) and compared using Kruskal‒Wallis’s equality-of-populations rank test. Categorical and ordinal variables were summarized using proportions and percentages and were compared using Pearson’s chi-square test or Fisher’s exact test, as appropriate. All analyses were performed using Stata version 15 (StataCorp LP, College Station, TX, USA). The level of significance was set at *p* < 0.05.

## Results

### Patient characteristics

We enrolled 28 participants with KF receiving lamivudine-based regimens and managed with CAPD. Participants had a median age of 42.6 (IQR, 38.9–48.1) years at the time of enrolment with a median body mass index (BMI) of 22.5 (IQR, 20.8–24.1) Kg/m^2^. More females were enrolled [71.4% (20/28)]. Most participants received lamivudine 50 mg (78.6%, 22/28). The median duration of ARV treatment was 8 (IQR, 4.5–10.5) years, and CAPD treatment was 13.3 (IQR, 3.3–31.9) months. The median T-cell CD4 count was 356 (IQR, 234–500) cells/mm^3^. The frequency of HIV-1 detectable in CAPD effluents was 14.3% (4/28) with median viral load in serum and CAPD effluents 43,400 (IQR, < 20–101000) and 388 (< 20–675) copies/mL, respectively. Furthermore, the majority (85.7%, 24/28) of participants had suppressed serum and CAPD viral loads. Lastly, the peritoneal membrane type and pharmacokinetic measures were variable among study participants who underwent PET, Table 1.

**Table 1:** Characteristics of the pharmacokinetic study population

Variables	Lamivudine dosages				Total (N = 28)
	50 mg (N = 22)	75 mg (N = 3)	300 mg (N = 3)	P-value	
Demographics					
Age (years), median (IQR)	42.6 (38.5–49.1)	41.4 (27.1–45.1)	43.8 (41.3–48.8)	0.673	42.6 (38.9–48.1)
Sex					
Female, *n*/*N* (%)	16/22 (72.7%)	3/3 (100%)	1/3 (33.3%)	0.187	20/28 (71.4%; 95% CI, 51.4–85.5%)
Ethnicity					
African, *n*/*N* (%)	22/22 (100%)	3/3 (100%)	3/3 (100%)		28/28 (100%)
Marital status					
Married, *n*/*N* (%)	4/22 (18.2%)	0/3 (0.0%)	1/3 (33.3%)	0.565	5/28 (17.9%; 95% CI, 7.3–37.4%)
Single, *n*/*N* (%)	18/22 (81.8%)	3/3 (100%)	2/3 (66.7%)		23/28 (82.1%; 95% CI, 62.5–92.7%)
Employment status					
Employed, *n*/*N* (%)	1/22 (4.6%)	0/3 (0.0%)	3/3 (100%)	0.868	1/28 (3.6%; 95% CI, 0.5–23.0%)
Unemployed, *n*/*N* (%)	21/22 (95.5%)	3/3 (100%)	3/3 (100%)		27/28 (96.4%; 95% CI, 76.9–99.5%)
Residential area					
Rural, *n*/*N* (%)	3/22 (13.6%)	1/3 (33.3%)	2/3 (66.7%)	0.096	6/28 (21.4%; 95% CI, 9.5–41.2%)
Township, *n*/*N* (%)	19/22 (86.4%)	2/3 (66.7%)	1/3 (33.2%)		22/28 (78.6%; 95% CI, 58.5–90.4%)
Highest education					
Primary school, *n*/*N* (%)	3/22 (13.6%)	0/3 (0.0%)	1/3 (33.3%)	0.856	4/28 (14.3%; 95% CI, 5.2–33.5%)
High school, *n*/*N* (%)	13/22 (59.1%)	2/3 (66.7%)	1/3 (33.3%)		16/28 (57.1%; 95% CI, 37.8–74.5%)
Grade 12 Certificate, *n*/*N* (%)	4/22 (18.2%)	1/3 (33.3%)	1/3 (33.3%)		6/28 (21.4%; 95% CI, 9.5–41.2%)
Degree, *n*/*N* (%)	2/22 (9.1%)	0/3 (0.0%)	0/3 (0.0%)		2/28 (7.1%; 95% CI, 1.7–25.7%)
Cigarette smoker, *n*/*N* (%)	0/22 (0.0%)	0/3 (0.0%)	1/3 (33.3%)	**0.013**	1/28 (3.6%; 95% CI, 0.5–23.0%)
Alcoholic, *n*/*N* (%)	2/22 (9.1%)	0/3 (0.0%)	0/3 (0.0%)	0.745	2/28 (7.1%; 95% CI, 1.7–25.7%)
Detection of HIV-1 by PCR					
HIV-1 Detection frequency					
Plasma, *n*/*N* (%)	7/22 (31.8%)	2/3 (66.7%)	1 (33.3%)	0.781	10/28 (35.7%; 95% CI, 19.8–55.5%)
CAPD effluents, *n*/*N* (%)	1/22 (4.6%)	2/3 (66.7%)	1 (33.3%)	0.022	4/28 (14.3%; 95% CI, 5.2–33.5%)
HIV-1 viral load					
Plasma HIV-1 viral load (copies/mL), median (IQR) *	229 (109–736)	72,200 (43,400–101000)	1980	0.184	653.5 (123–18,100)
CAPD HIV-1 viral load (copies/mL), median (IQR) *	33	531.5 (388–675)	30	0.007	210.5 (31.5–531.5)
Clinical characteristics					
Diabetes, *n*/*N* (%)	0/22 (0.0%)	0/3 (0.0%)	0/3 (0.0%)		0/28 (0.0%)
Hypertension, *n*/*N* (%)	21/22 (96.2%)	3/3 (100%)	3/3 (100%)	0.868	27/28 (96.4%; 95% CI, 76.8–99.5%)
< 1 year, *n*/*N* (%)	0/22 (0.0%)	1/3 (33.3%)	0/3 (0.0%)	0.142	1/28 (3.6%; 95% CI, 0.5–23.0%)
1–5 years, *n*/*N* (%)	10/22 (45.5%)	1/3 (33.3%)	1/3 (33.3%)		12/28 (42.9%; 95% CI, 25.5–62.1%)
5–10 years, *n*/*N* (%)	5/22 (22.7%)	1/3 (33.3%)	2/3 (66.7%)		8/28 (28.6%; 95% CI, 14.5%-48.6%)
> 10 years, *n*/*N* (%)	6/22 (27.3%)	0/3 (0.0%)	0/3 (0.0%)		6/28 (21.4%)
Non-hypertensive, *n*/*N* (%)	1/22 (4.6%)	0/3 (0.0%)	0/3 (0.0%)		1/28 (3.6%; 95% CI, 0.4–23.0%)
ARV duration (years), median (IQR)	8 (5–11)	6 (2–16)	8 (3–9)	0.849	8 (4.5–10.5)
Lamivudine drug					
FDC, *n*/*N* (%)	0/22 (0.0%)	0/3 (0.0%)	3/3 (0.0%)	**0.001**	3/28 (10.7%)
Solution, *n*/*N* (%)	22/22 (100%)	0/3 (0.0%)	0/3 (0.0%)		22/28 (78.6%)
Tablet, *n*/*N* (%)	0/22 (0.0%)	3/3 (100%)	0/3 (0.0%)		3/28 (10.7%)
CAPD duration (months), median (IQR)	13.3 (3.4–34.5)	3.2 (1.8–17.7)	29.3 (6.0–62.3)	0.304	13.3 (3.3–31.9)
< 6 (months), *n*/*N* (%)	7/22 (31.8%)	2/3 (66.7%)	1/3 (33.3%)	0.390	10/28 (35.7%)
6–2 (years), *n*/*N* (%)	9/22 (40.9%)	1/3 (33.3%)	0/3 (0.0%)		10/28 (35.7%)
2–5 (years), *n*/*N* (%)	5/22 (22.7%)	0/3 (0.0%)	1/3 (33.3%)		6/28 (21.4%)
> 5 (years), *n*/*N* (%)	1/22 (4.6%)	0/3 (0.0%)	1/3 (3.3%)		2/28 (7.1%)
PD system					
Dianeal (Baxter), *n*/*N* (%)	20/22 (90.9%)	3/3 (100%)	1/3 (33.3%)	**0.021**	24/28 (85.7%; 95% CI, 66.5–94.8%)
Stay safe (Fresenius), *n*/*N* (%)	2/22 (9.1%)	0/3 (0%)	2/3 (66.7%)		4/28 (14.3%; 95% CI, 5.2–33.5%)
PD system strength					
Glucose 1.5%, *n*/*N* (%)					
Dwell 1	1/22 (4.6%)	0/3 (0%)	0/3 (0%)	0.825	1/28 (3.6%; 95% CI, 0.04–23.0%)
Dwell 2	5/22 (22.7%)	1/3 (33.3%)	0/3 (0%)		6/28 (21.4%; 95% CI, 9.6–41.2%)
Dwell 3	4/22 (18.2%)	0/3 (0%)	0/3 (0%)		4/28 (14.3%; 95% CI, 5.2–33.5%)
Dwell 4	10/22 (45.5%)	2/3 (66.7%)	3/3 (100%)		(53.6%; 95% CI, 34.6–71.5%)
Glucose 2.5%, *n*/*N* (%)					
Dwell 1	3/22 (13.6%)	0/3 (0%)	0/3 (0%)	0.823	3/28 (10.7%;95% CI, 3.3–29.6%)
Dwell 2	6/22 (27.3%)	1/3 (33.3%)	0/3 (0%)		7/28 (25%; 95% CI, 11.9–44.9%)
Dwell 4	1/22 (4.6%)	0/3 (0%)	0/3 (0%)		1/28 (3.6%; 95% CI, 0.5–23.0%)
Glucose 4.25%, n/N (%)	3/22 (13.6%)	0	0		3/28 (10.7%; 95% CI, 3.3–29.6%)
Ecodextrin, *n*/*N* (%)	2/22 (9.1%)	0	0		2/28 (7.1%; 95% CI, 1.7–25.7%)
PET UF (ml), median (IQR)	300 (300–400)	300 (300–300)	400 (400–400)	0.415	300 (300–400)
PET D/P Creatinine, median (IQR)	0.65 (0.57–0.74)	0.72 (0.64–0.79)	0.71 (0.69–0.72)	0.631	0.68 (0.60–0.74)
PET D/D_0_ Glucose, median (IQR)	0.47 (0.40–0.58)	0.52 (0.50–0.54)	0.47 (0.40–0.54)	0.864	0.50 (0.40–0.55)
Peritoneal membrane type					
Low-average transporter, *n*/*N* (%)	8/16 (50%)	1/2 (50%)	0/2 (0.0%)	0.777	9/20 (45%; 95% CI, 24.2–67.7%)
High-average transporter, *n*/*N* (%)	7/16 (43.8%)	1/2 (50%)	2/2 (100%)		10/20 (50%; 95% CI, 28.2–71.8%)
High transporter, *n*/*N* (%)	1/16 (6.3%)	0/2 (0.0%)	0/2 (0.0%)		1/20 (5%; 95% CI, 0.6–31.1%)
Temperature (°C), median (IQR)	36.9 (36.7–37)	37 (36.7–37.1)	37 (37–37.3)	0.050	36.9 (36.7–37)
Systolic BP (mmHg), median (IQR)	159.5 (122–168)	170 (169–180)	139 (128–140)	0.132	159.5 (129–169.5)
Diastolic BP (mmHg), median (IQR)	87 (79–93)	101 (101–106)	85 (80–95)	0.119	87 (80–98.5)
Weight (Kg), median (IQR)	58.9 (53.9–66.3)	61.3 (47.6–63.3)	62.6 (49.2–73.5)	0.899	59 (52.1–64.8)
BMI (Kg/m^2^), median (IQR)	22.6 (20.4–23.9)	22.2 (21.3–26.3)	22.4 (18.5–33.4)	0.994	22.5 (20.8–24.1)
Laboratory parameters					
White cell count (× 10^9^/L), median (IQR)	33.75 (32–35)	36.5 (35–37.5)	34 (32–36.3)	0.235	34 (32–36.3)
T-cell CD4 count (cells/µL), median (IQR)	353 (250–490)	515 (167–863)	400 (187–561)	0.993	356 (234–500)
HIV PD effluents viral load (copies/mL), median (IQR)	< 20 (< 20)	388 (< 20–675)	< 20 (< 20–30)		< 20 (< 20)
HIV Plasma viral load (copies/mL), median (IQR)	< 20 (< 20–109)	43,400 (< 20–101000)	< 20 (< 20–1980)		< 20 (< 20–176)
Haemoglobin (g/dL), median (IQR)	11.95 (9.7–12.6)	9.8 (7.8–9.8)	11.5 (11.1–13)	0.186	11.5 (9.8–12.6)
Sodium (mmol/L), median (IQR)	141 (137–143)	136 (135–139)	139 (135–142)	0.429	140 (135–143)
Potassium (mmol/L), median (IQR)	3.9 (3.5–4.4)	4.1 (3–4.2)	4.3 (3.9–6.4)	0.383	4 (3.5–4.4)
Urea (mmol/L), median (IQR)	19.6 (15–23.1)	13.4 (9.2–23.4)	24.3 (10.7–36.7)	0.518	19.6 (12.4–23.9)
Creatinine (µmol/L), median (IQR)	823.5 (706–1092)	474 (11.7–750)	612 (326–711)	0.042	746.5 (630.5–1037
Calcium (mmol/L), median (IQR)	2.2 (2.0–2.2)	2.2 (2.1–2.6)	1.9 (1.6–2.2)	0.337	2.2 (2.0–2.2)
Phosphate (mmol/L), median (IQR)	1.2 (1.0–1.7)	1.4 (0.9–2.0)	1.3 (0.9–1.4)	0.856	1.3 (0.9–1.7)
Albumin (g/L), median (IQR)	28 (24–30)	23 (21–29)	20 (17–32)	0.321	26.5 (22.5–29.5)
Ferritin (µg/L), median (IQR)	290.5 (162–605)	244 (188–342)	388 (287–407)	0.744	312 (195.6–516)

*ARV* antiretroviral, *BMI* body mass index, *CAPD* continuous ambulatory peritoneal dialysis, *D/P* dialysate to plasma ratio, *FDC* fixed-dosage combination, *PD* peritoneal dialysis, *PET* peritoneal equilibration test, *UF* ultrafiltration

^*^HIV viral load among those with detectable HIV-1 PCR

### Safety

Lamivudine and CAPD procedures were well tolerated among the study population. No adverse reactions were observed during the three-day admission.

### Steady-state serum lamivudine pharmacokinetic analysis

#### 50 mg oral solution lamivudine pharmacokinetic measures

The observed geometric mean of lamivudine AUC (AUC_0-24_) was higher (666.04 ± 434.8 vs 341.76 ± 0 ng/mL) among the undetectable CAPD HIV-1 viral load cohort when compared to a cohort with detectable HIV-1 in CAPD. T_1/2_ was decreased (10.47 ± 0 vs 18.70 ± 9.0 h per hour) in the detectable cohort of HIV-1 in CAPD than in a cohort with undetectable HIV-1 in CAPD effluents. The K_e_ was increased (0.07 vs 0.04) among the HIV-1 detectable cohort compared to the undetectable cohort. The C_max_ (42.03 ± 23.4 vs 30.2 ± 0 ng/mL) and C_min_ (18.3 ± 13.7 vs 6.7 ± 0 ng/mL) were increased in a cohort undetectable of HIV-1 in CAPD than a detectable cohort. However, T_max_ among the two groups was comparable (4.05 ± 2.2 vs 4 ± 0 h); although it was lower compared to T_max_ in the peritoneal compartment (11.81 ± 4.5 vs 12 ± 0 h), Table 2.

**Table 2:** Steady-state lamivudine pharmacokinetic measure among different dosage groups of participants with detectable and undetectable HIV viral load in CAPD effluents

PK measure	HIV PD undetectable			HIV PD Detectable			Total		
	50 mg (N = 21)	75 mg (N = 1)	300 mg (N = 2)	50 mg (N = 1)	75 mg (N = 2)	300 mg (N = 1)	50 mg (N = 22)	75 mg (N = 3)	300 mg (N = 3)
PK measure in blood serum									
AUC_0-24 h_ (ng/mL/hr), mean (± SD)	666.04 (± 434.8)	103.67	3814.33 (± 836.8)	341.76	964.93 (± 628.0)	1779.02	651.3 (± 429.9)	677.84 (± 666.7)	3135.89 (± 1315.6)
AUC_0-∞_ (ng/mL/hr), mean (± SD)									
AUC_Line (ng/mL/hr), mean (± SD)	914.05 (± 765.8)	163.83	4568.99 (± 814.2)	373.39	1082.20 (± 757.5)	2208.20	889.47 (± 756.1)	776.08 (± 753.7)	3782.06 (± 1479.6)
AUC_Exp (ng/mL/hr), mean (± SD)	1264.27 (± 1209.8)	241.63	5824.56 (± 892.3)	441.15	1323.16 (± 951.5)	2877.19	1226.86 (± 1193.7)	962.65 (± 917.9)	4842.11 (± 1814.9)
AUC_log (ng/mL/hr), mean (± SD)	1277.56 (± 1249.8)	239.67	5835.09 (± 920.1)	443.26	1333.21 (± 942.8)	2868.94	1239.64 (± 1232.1)	968.7 (± 918.2)	4846.37 (± 1831.9)
T_1/2_, mean (± SD)	18.70 (± 9.0)	28.40	15.41 (± 2.3)	10.47	10.03 (± 4.3)	16.86	18.32 (± 9.0)	16.15 (± 11.0)	15.9 (± 1.8)
K_e_ constant (per hour per mL)	0.04	0.02	0.05	0.07	0.08	0.04	0.04	0.06	0.04
C_max_, mean (± SD)	42.03 (± 23.4)	6.04	245.20 (± 15.7)	30.20	76.80 (± 33.9)	107.00	41.49 (± 22.9)	53.21 (± 47.4)	199.13 (± 95.7)
T_max_ (hrs), mean (± SD)	4.05 (± 2.2)	2.00	3.00 (± 1.7)	4.00	7.00 (± 7.1)	4.00	4.05 (± 2.1)	4.67 (± 6.4)	3.33 (± 1.2)
C_min_ (ng/mL), mean (± SD)	18.3 (± 13.7)	3.3	92.2 (± 17.5)	6.7	22.9 (± 12.0)	44.8	17.8 (± 13.6)	16.4 (± 14.1)	76.4 (± 30.0)
PK measure in CAPD effluents									
AUC_0-24 h_ (ng/mL/hr), mean (± SD)	398.14 (± 264.3)	72.38	2363.30 (± 415.4)	107.12	538.67 (± 378.9)	1278.20	384.91 (± 265.3)	383.24 (± 379.9)	2001.60 (± 691.9)
AUC_0-∞_ (ng/mL/hr), mean (± SD)									
AUC_Line	1210.07 (± 2529.7)	111.73	3401.41 (± 271.3)	114.00	648.63 (± 354.4)	1508.24	1157.88 (± 2477.2)	469.66 (± 398.6)	2770.35 (± 1109.7)
AUC_Exp	2100.67 (± 5100.7)	165.00	4870.61 (± 140.3)	134.32	862.62 (± 386.3)	1926.24	2007.03 (± 4989.9)	630.08 (± 486.7)	3889.16 (± 1702.8)
AUC_log	2235.44 (± 5768.1)	164.97	4759.65 (± 183.6)	133.99	879.25 (± 408.9)	1933.31	2135.37 (± 5640.7)	641.15 (± 503.6)	3817.54 (± 1636)
T_1/2_ (hrs), mean (± SD)	176.02 (± 614.7)	26.96	22.86 (± 6.8)	7.57	14.81 (± 7.3)	13.76	168.00 (± 600.3)	18.86 (± 8.7)	19.83 (± 7.1)
K_e_ constant (per hour per mL)	0.05	0.03	0.03	0.09	0.05	0.05	0.05	0.04	0.04
C_max_ (ng/mL), mean (± SD)	22.72 (± 14.4)	3.78	132.50 (± 17.4)	7.38	39.20 (± 31.7)	65.60	22.02 (± 14.3)	27.39 (± 30.3)	110.20 (± 40.5)
T_max_ (hrs), mean (± SD)	11.81 (± 4.5)	6.00	12.00 (± 8.5)	12.00	12.00	4.00	11.82 (± 4.4)	10.00 (± 3.5)	9.33 (± 7.6)
C_min_ (ng/mL), mean (± SD)	13.2 (± 9.5)	2.4	73.2 (± 3.7)	2.5	18.5 (± 10.6)	33	12.7 (± 9.6)	13.2 (± 11.9)	59.8 (± 23.4)

*AUC*_*0-24 h*_ Area under the time-versus-concentration curve from 0 till 24-h period, *AUC*_*-Line*_ the area under the time-versus-concentration curve extrapolated by fitting a least-squares linear fit through the last few points, *AUC*_*_Exp*_ the area under the time-versus-concentration curve extrapolated by fitting a decreasing exponential curve through the last few data points, *AUC*_*_log*_ an area under the time-versus-concentration curve extrapolated by fitting a least-squares linear regression line on the log concentration, *T*_*1/2*_ half-life time at which drug dosage is reduced by 50% of its maximum dosage, *K*_*e*_ elimination constant rate, *C*_*max*_ maximum serum/CAPD effluent drug concentration, *T*_*max*_ time of maximum concentration, *C*_*min*_ minimum serum/CAPD effluent concentrations, *T*_*24*_ time of last concentration point observation, *PK* pharmacokinetics, *PD* peritoneal dialysis

#### 75 mg oral tablet lamivudine pharmacokinetic measures

**Fig. 1:**
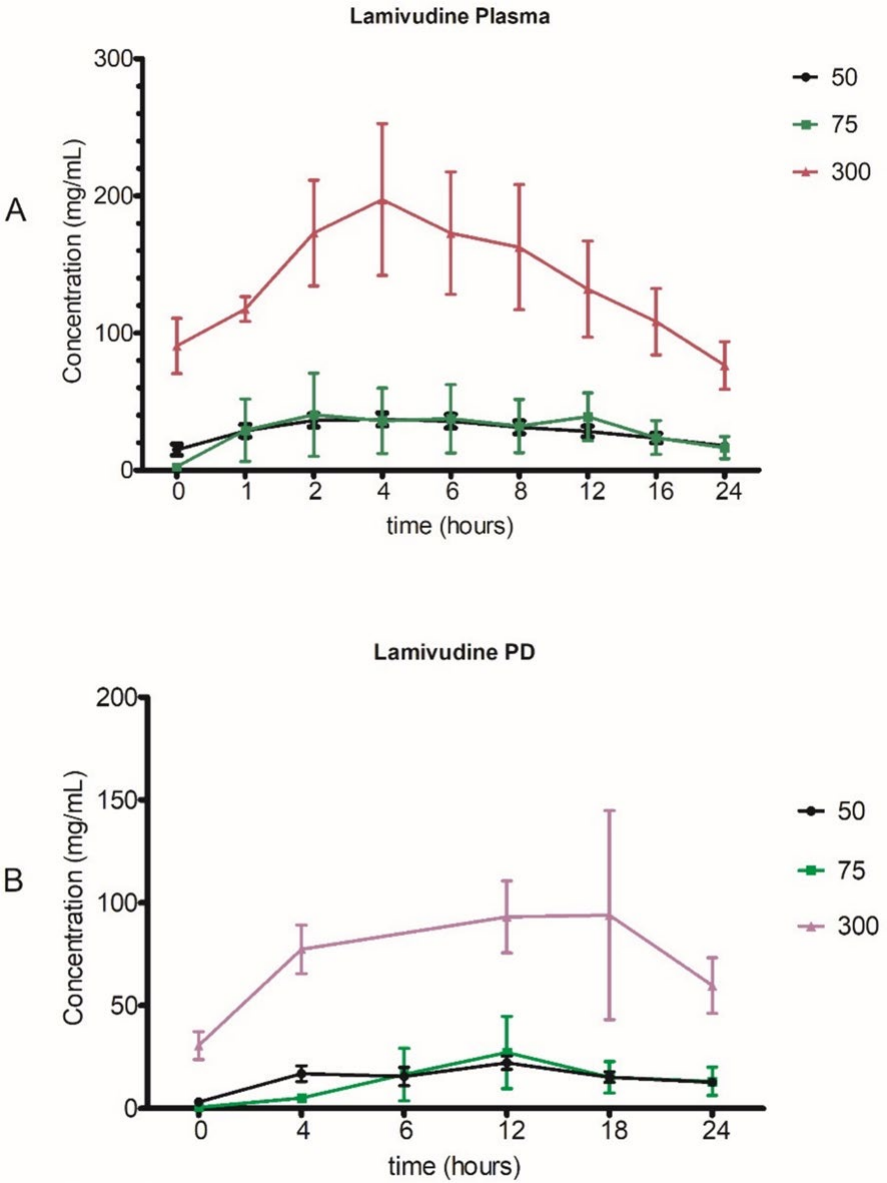
Concentration–time curve for lamivudine in blood serum and CAPD effluents. **A** shows the concentration time curve of lamivudine in blood serum and (**B**) in CAPD effluents over a period of 24 h

Lamivudine exposure in serum was higher, AUC_0-24_ (964.93 ± 628.0 vs 103.67 ± 0 ng/mL), and C_max_ (76.80 ± 33.9 vs 6.04 ± 0 ng/mL) in a cohort of HIV detectable in CAPD than an undetectable cohort. T_1/2_ was decreased (10.03 ± 4.3 vs 28.40 ± 0 h) in a cohort with detectable HIV in CAPD compared to the undetectable cohort. Alternatively, the rate of elimination was increased (0.08 vs 0.05) in the detectable than undetectable cohort, Table 2. The total blood serum exposure of lamivudine between participants who received 50 mg and 75 mg was comparable, Fig. 1.

#### 300 mg oral FDC lamivudine pharmacokinetics measures

The mean serum lamivudine exposure was increased in a cohort undetectable of HIV-1 in CAPD, AUC_0-24_ (3814.33 ± 836.8 vs 1779.02 ± 0 ng/mL) than in a detectable cohort. The K_e_ (0.05 vs 0.04) and T_1/2_ (15.41 ± 2.3 vs 16.86 ± 0) were almost comparable among the two cohorts. Although the T_max_ (3 ± 1.7 vs 4 h ± 0) was comparable, the C_max_ (245.20 ± 15.7 vs 107.00 ± 0 ng/mL) and C_min_ (92.2 ± 17.5 vs 44.8 ± 0 ng/mL) was variable and increased among a cohort of undetectable HIV-1 in CAPD than the detectable cohort, Table 2. All participants were over-exposed to lamivudine at 300 mg compared to 50 and 75 mg dosages, Fig. 1.

### Steady-state lamivudine pharmacokinetics in the peritoneal compartment

#### 50 mg lamivudine oral solution pharmacokinetic measures

The lamivudine exposure in the peritoneal cavity was increased in a cohort undetectable of HIV-1 in CAPD effluents, AUC_0-24_ (398.14 ± 264.3 vs 107.12 ± 0 ng/mL), and C_max_ (22.72 ± 14.4 vs 7.38 ± 0 ng/mL) compared to a cohort detectable of HIV in CAPD. T_1/2_ was decreased (7.57 ± 0 vs 176.02 ± 614.7 h) in a cohort detectable of HIV-1 in CAPD than undetectable cohort. Alternatively, k_e_ was increased (0.09 vs 0.05) in a cohort detectable of HIV-1 in CAPD than undetectable cohort, Table 2.

#### 75 mg lamivudine oral tablet pharmacokinetic measures

The exposure of lamivudine in the peritoneal cavity was decreased in a cohort undetectable of HIV-1 in CAPD effluents, AUC_0-24_ (72.38 ± 0 vs 538.67 ± 378.9 ng/mL), and C_max_ (3.78 ± 0 vs 39.20 ± 31.7 ng/mL) compared to a cohort detectable of HIV-1 in CAPD effluents. T_1/2_ was lower (14.2 ± 7.3 vs 26.96 ± 0) in a cohort detectable of HIV-1 in CAPD. However, k_e_ was higher (0.05 vs 0.03) in the CAPD HIV-1 detectable cohort than the undetectable cohort, Table 2.

#### 300 mg lamivudine FDC oral table pharmacokinetic measures

The mean lamivudine exposure was lower in a cohort detectable of HIV-1 in CAPD effluents, AUC_0-24_ (1278.20 ± 0 vs 2363.30 ± 415.4 ng/mL) and C_max_ (65.60 ± 0 vs 132.50 ± 17.4 ng/mL) with a decreased T_max_ (4.00 ± 0 vs 12.00 ± 8.5 h) compared to the undetectable cohort. Although the apparent T_1/2_ was decreased (13.76 ± 0 vs 22.86 ± 6.8 h) in a cohort detectable of HIV-1 in CAPD, the k_e_ was increased (0.05 vs 0.03) compared to a cohort undetectable of HIV-1 in CAPD, Table 4.

## Discussion

This study evaluated the steady-state lamivudine pharmacokinetics in KF patients with suppressed and unsuppressed CAPD viral load. The blood serum and CAPD pharmacokinetic measures were variable among cohorts with detectable and undetectable HIV-1 in CAPD effluents. The total exposure of lamivudine was higher in blood compared to CAPD effluents for all lamivudine dosages. Serum and CAPD exposure was decreased in two participants with detectable CAPD viral load and treated with 50 and 300 mg, respectively; however, it was within the therapeutic ranges in serum for all participants.

Lamivudine serum exposure (AUC_0-24_) was variable among participants (4/28) shedding HIV-1 in CAPD effluents, possibly due to inter-patient pharmacokinetic variability. Furthermore, lamivudine concentrations, including C_min_, in these participants, were above the reported lamivudine 90% inhibitory concentration (IC_90_) range against HIV-1 in various cell lines (0.0087 to 0.464 µg/ml) [15]; thus, suggesting adequate therapeutic exposure. Studies on steady-state lamivudine pharmacokinetics by Bohjanen et al. [6] reported the serum AUC_0-24_ (49,800 ng/h/mL), C_max_ (3770 ng/mL), and C_min_ (1410 ng/mL) in HIV-suppressed CAPD participants treated with a daily dosage of 150 mg; furthermore, Yuen et al. [15] reported AUC_0-24_ (9210 ng/h/mL) and C_max_ (1190 ng/mL) and C_min_ (90 ng/mL) in steady-state non-KF patients who received 150 mg bi-daily dosage. A study by Heald et al. [7] looked at non-steady-state KF patients receiving 300 mg lamivudine dosage and reported a C_max_ of 5684 ng/mL. These mean concentration values were also above the reported IC_90_ and IC_50_ [16–18] and within a comparable range to our findings. The concentrations shown in Bohjanen study [6] were effective and tolerated and suggested a reduced daily dosage of 25 mg in dialysis patients which is suggested to provide the same exposure to 150 mg bi-daily dosage in non-KF patients. This may suggest 50 mg, 75 mg and 300 mg daily dosages to be effective in our study population. However, safety studies may be necessary to evaluate the tolerability of these dosages.

The T_max_ in participants shedding HIV-1 in CAPD effluents was almost comparable to participants with suppressed HIV-1 in CAPD. This was an expected finding, since impaired renal function does not affect lamivudine absorption in the gastrointestinal tract. Furthermore, this may suggest an almost comparable absorption rate of lamivudine into the systemic circulation and penetrance into CAPD effluents among these participants and the majority with a suppressed HIV-1 viral load.

The rate of elimination in participants shedding HIV-1 in CAPD effluents was slightly increased in CAPD effluents (5–9% per hour per mL) compared to serum (4–8% per hour per mL) samples among all lamivudine dosages. The elimination rate in CAPD effluents was almost half-fold lower compared to 16% reported in a study by Bohjanen et al. [6]. Notably, a patient who received 50 mg and shedding HIV-1 in CAPD effluents had an increased elimination rate in the PD compartment (9%) with a decreased half-life (7.6 h) compared to the majority of patients who were HIV-suppressed. Although the hypertonic dialysate solution and peritoneal membrane type are hypothesised to influence drug elimination in CAPD effluents [19, 20], this was not the case in this patient because the elimination rate could not reduce the AUC_0-24_ and C_max_ and C_min_ (6.7 ng/mL) below the therapeutic levels, and was not below C_min_ reported as 1410 ng/mL in [6]. Thus, it may not affect the bioavailable lamivudine pro-drug effective concentrations in the serum.

The steady-state lamivudine exposure in serum and CAPD samples does not suggest inadequate lamivudine dosing across all study participants (Table 2). Lamivudine is maintained even in 'renal-friendly' ART or second-line regimens due to its ability to enhance the susceptibility/pharmacodynamics of NRTI, Zidovudine [21], and Tenofovir [22] against HIV drug resistance mutations. However, viral suppression is unlikely in the presence of M184V mutation. HIV-1 shedding among participants who received a higher lamivudine dosage did not alter pharmacokinetics parameters. Thus, suggesting HIV drug resistance mutation may be possible influencers of HIV-1 shedding in CAPD effluents as demonstrated in a cross-sectional study by Mooko et al. [12].

This study had several limitations, first, this sub-study was not powered for pharmacokinetic analysis of 75 mg and 300 mg lamivudine doses. Second, our study did not quantify the active lamivudine metabolite, 5′-triphosphate in study samples as it is only generated from a small (30%) fraction of lamivudine pro-drug, which undergoes a minor route excretion.

## Conclusions

The lamivudine dosages evaluated in this study do not suggest inadequate dosing in KF patients managed with CAPD even in those shedding HIV-1 in CAPD effluents. Thus, highlighting a need for improved ARV treatment stratagems to mitigate challenges associated with adherence and intensify health education in this patient population.

## Data Availability

Not applicable.
